# Upadacitinib in Biologic-Naïve Patients With Acute Severe Ulcerative Colitis: A Case Report of a Promising Alternative Rescue Therapy

**DOI:** 10.7759/cureus.110365

**Published:** 2026-06-06

**Authors:** Sneh Parekh, Antoine Boustany, Yasasvhinie Santharam, Claudia Aleman Oliva, Camille Lupianez, Andrew T Flint, Scott Smith, Marianny Sulbaran

**Affiliations:** 1 Internal Medicine, University of Florida College of Medicine – Jacksonville, Jacksonville, USA; 2 Gastroenterology, University of Florida College of Medicine – Jacksonville, Jacksonville, USA; 3 Pathology, University of Florida College of Medicine – Jacksonville, Jacksonville, USA

**Keywords:** acute severe ulcerative colitis, inflammatory bowel disease, jak inhibitors, ulcerative colitis, upadacitinib

## Abstract

Acute severe ulcerative colitis (ASUC) is a known complication of ulcerative colitis (UC). Treatment of ASUC is with intravenous (IV) corticosteroids as first-line therapy and with infliximab or cyclosporine as rescue therapy in disease refractory to steroids. Upadacitinib is a newer and alternative therapy for patients, particularly after they have received at least one approved systemic therapy, if tumor necrosis factor (TNF) blockers are clinically inadvisable. We present a case of a biologic-naïve hospitalized patient with corticosteroid-refractory ASUC, who was not initially treated with TNF inhibitor therapy, yet who achieved excellent outcomes with upadacitinib salvage therapy. Upadacitinib can have potential benefits as an alternative rescue therapy in the management of select ASUC cases. Though rescue therapy options exist, patients often still require surgical management, requiring further alternative treatments for ASUC. Upadacitinib can serve as a promising treatment for ASUC, with new expanded indications further justifying its utility.

## Introduction

Acute severe ulcerative colitis (ASUC) is a life-threatening complication experienced by 25% of patients with ulcerative colitis (UC) [1]. The American College of Gastroenterology (ACG) and American Gastroenterological Association (AGA) define ASUC in a hospitalized patient with ≥ 6 bloody bowel movements per day with one additional marker of systemic toxicity, based on the Truelove and Witts criteria [1,2]. Despite advances in medical therapy for inflammatory bowel disease, ASUC still carries a mortality risk, and colectomy rates remain high [1].

The AGA recommends using intravenous (IV) corticosteroids for three to five days as first-line therapy, with infliximab or cyclosporine thereafter for steroid-refractory disease [2]. More recently, case series have demonstrated the efficacy of the off-label use of Janus kinase (JAK) inhibitors as rescue therapy in biologic-experienced hospitalized patients with ASUC [2,3].

A recent Food and Drug Administration (FDA)-updated indication statement now allows the use of upadacitinib for patients after they have received at least one approved systemic therapy in the event that tumor necrosis factor (TNF) blockers are clinically inadvisable. However, the implications of this statement in the management of patients with ASUC are yet to be determined [4,5]. Here, we present a case of a biologic-naïve hospitalized patient with steroid-refractory ASUC who achieved excellent outcomes with salvage therapy using upadacitinib.

This article was previously presented as a meeting abstract at the 2025 Advances in Inflammatory Bowel Diseases Conference on December 8, 2025.

## Case presentation

We present the case of a 31-year-old male with a past medical history of a positive hepatitis B surface antigen, who was biologic-naïve, and was previously diagnosed with left-sided ulcerative colitis at the age of 21. During the patient's initial flare at that time, he was treated with an oral prednisone taper, followed by mesalamine for two years, after which he achieved clinical remission. However, mesalamine was then self-discontinued, and he was lost to follow-up.

The patient presented to our gastroenterology clinic with complaints of abdominal pain and decreased appetite, a 12-pound weight loss over two weeks, and 5-15 bloody stools per day. Physical examination was notable for an abdomen that was tender to deep palpation. A colonoscopy revealed mucosal bleeding and ulceration, along with scattered erosions in the terminal ileum that were felt to represent backwash ileitis (Figure 1), graded as Mayo Score 3 in severity.

**Figure 1:**
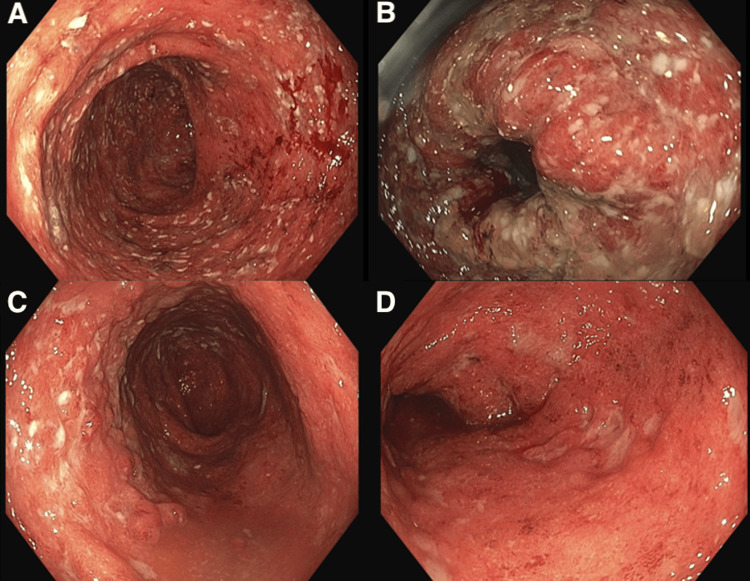
Colonoscopy showing pancolitis graded as a Mayo Score 3 in severity, along with spontaneous bleeding and ulcerations in the mucosa Sigmoid colon (a), transverse colon (b), ascending colon (c), and rectum (d)

Biopsies demonstrated acute on chronic colitis with acute cryptitis, crypt abscesses, and architectural distortion throughout the colon, with no dysplasia or granulomas identified (Figure 2).

**Figure 2:**
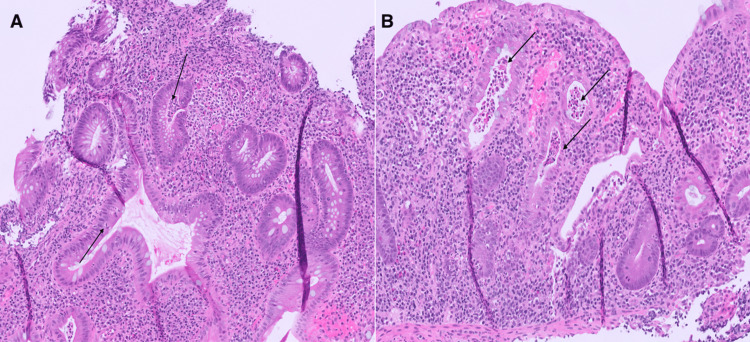
Biopsies revealing acute-on-chronic colitis with acute cryptitis, crypt abscesses, and architectural distortion throughout the colon (black arrows) Ascending colon (a) and descending colon (b)

Laboratory workup was significant for a significantly elevated C-reactive protein (CRP) level, white blood cell (WBC) count, and erythrocyte sedimentation rate (ESR), with a decreased hemoglobin level (Table 1). The patient met the criteria for ASUC and was subsequently hospitalized.

**Table 1:** Relevant laboratory testing obtained Note that erythrocyte sedimentation rate (ESR) values were not trended, as in the management of acute severe ulcerative colitis (ASUC), and C-reactive protein (CRP) levels respond faster to acute inflammatory changes and are more reliable when assessing treatment response, compared to ESR.

Relevant laboratory testing	Values on presentation	Values on hospital day 4 (after initiation of upadacitinib)	Values on discharge	Reference ranges
C-reactive protein (CRP)	226 mg/L	66 mg/L	3.97 mg/L	0.1-2.8 mg/L
White blood cell (WBC) count	14.26 x 10^9^ cells/L	6.61 x 10^9^ cells/L	12.89 x 10^9^ cells/L	4.5-11 x 10^9^ cells/L
Hemoglobin	12.6 g/dL	11.0 g/dL	11.9 g/dL	14-18 g/dL
Erythrocyte sedimentation rate (ESR)	56 mm/hour	-	-	0-15 mm/hour
Albumin	4.0 g/dL	3.0 g/dL	3.6 g/dL	3.8-4.9 g/dL

The patient tested negative for *Clostridium difficile* infection. Imaging with a magnetic resonance enterography (MRE) demonstrated active inflammation, consistent with an ulcerative colitis flare, without evidence of bowel obstruction or an intra-abdominal fluid collection (Figure 3).

**Figure 3:**
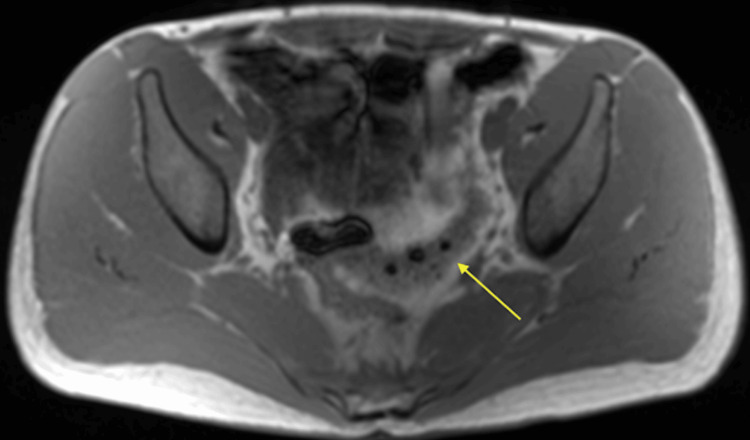
Magnetic resonance enterography (MRE) demonstrating continuous restricted diffusion, homogeneous bowel wall thickening, and submucosal enhancement in the rectosigmoid colon (yellow arrow)

The patient was started on IV methylprednisolone 20 mg, three times daily. Despite this, on hospital day three, his CRP remained significantly elevated, and he met criteria for initiation of rescue therapy. Due to his positive hepatitis B surface antigen, there was a comparable risk of viral reactivation with both TNF inhibitor and JAK inhibitor therapy. Given that he remained steroid-refractory and with severe disease, as evidenced by the hypoalbuminemia due to his increased intestinal permeability, the decision was made to start him on oral upadacitinib 30 mg twice daily, as supported by a recent case series, along with concomitant tenofovir 300 mg daily [6]. The goal of the decision to proceed with upadacitinib was to achieve rapid remission and symptomatic improvement and to prevent the risk of worsening disease progression that could lead to total colectomy if the patient were to potentially fail anti-TNF therapy [6].

Thereafter, his leukocytosis and CRP would improve within 24 hours, with a greater than 20% decrease in CRP levels since initiation of upadacitinib (Table 1), along with resolution of his abdominal pain and bloody stools. On the day of discharge, his CRP levels had nearly normalized, his hemoglobin levels had stabilized without complaints of bloody stools, and his white blood cell count, despite fluctuations that are to be expected during acute flares, was marginally above the expected reference range (Table 1). He was ultimately discharged to complete a five-week prednisone taper and to continue upadacitinib 45 mg per day. Four weeks after discharge, he was still completing upadacitinib induction along with the prednisone taper, and he was having Bristol scale 4 stools, without blood or complaints of abdominal pain. However, treat-to-target markers were not yet available, as he had not yet completed repeat inflammatory marker testing. Unfortunately, the patient experienced health insurance barriers, was unable to complete a follow-up colonoscopy, and was ultimately lost to follow-up.

## Discussion

Both the AGA and ACG recommend administering IV methylprednisolone for three to five days to reduce the risk of colectomy in these patients after concomitant infections such as *C. difficile* have been ruled out, along with rescue therapy with either infliximab or cyclosporine if patients are refractory to IV steroids [2,7]. This recommendation is supported by moderate-quality evidence for infliximab and low-quality evidence for cyclosporine [2,7]. However, despite the use of these rescue therapy options, patients may still require a colectomy, bringing attention to necessary alternatives in the treatment of ASUC.

Data from the U-ACHIEVE and U-ACCOMPLISH phase 3 trials showed that upadacitinib at a dose of 45 mg daily significantly improved remission rates compared to placebo, with 26% of patients achieving remission in the U-ACHIEVE (UC1) trial and 34% in the U-ACCOMPLISH (UC2) trial, versus 5% and 4% in the placebo groups, respectively [6,8]. Following these two trials, one post-hoc analysis revealed that upadacitinib 45 mg daily provided rapid relief from UC symptoms within the first few days of induction, along with reductions in inflammatory markers by the second week [9]. Another post-hoc analysis examined upadacitinib's impact on abdominal pain, urgency, and fatigue, showing that most patients reported no abdominal pain or bowel urgency from the second week, with these effects sustained through 52 weeks of maintenance therapy [8].

Some case series also show its effectiveness in the management of ASUC, with similar 90-day colectomy rates compared to infliximab or cyclosporine, and with a favorable safety profile [6]. Berinstein et al. showed that several biologic-naïve patients preferred to be treated with upadacitinib in the setting of ASUC [6].

Finally, recently updated FDA indications now allow for the use of upadacitinib after patients have received at least one approved systemic therapy in the event TNF blockers are clinically inadvisable [4]. The implications of this expanded indication have not yet been specifically analyzed in ASUC patients.

## Conclusions

This case highlights the potential benefit of upadacitinib as an alternative rescue therapy in the management of select cases of ASUC. Current FDA expanded indications may further facilitate its use in select cases. However, limitations include the lack of follow-up to assess long-term mucosal healing and corticosteroid-free remission, given the patient's insurance barriers. Continued research studies on the growing utility of upadacitinib are necessary to further expand its role in the management of ASUC, as it may provide a safe and well-tolerated therapeutic option in the management of this disease.
